# Form follows function: morphology as a map of mechanisms in neurodegenerative disease pathology

**DOI:** 10.17879/freeneuropathology-2026-9241

**Published:** 2026-01-21

**Authors:** Edward B. Lee

**Affiliations:** 1 Center for Neurodegenerative Disease Research, Institute on Aging, Department of Pathology and Laboratory, Medicine, Perelman School of Medicine, University of Pennsylvania, Philadelphia, Pennsylvania, USA

**Keywords:** Inclusion, Aggregate, Proteostasis, Transport, Tau, Amyloid

## Abstract

Across neurodegenerative diseases, the shape and spatial organization of pathology carry rich mechanistic information. Vacuoles, spongiosis, oligodendroglial coiled bodies, dendritic dystrophic neurites, amyloid plaque compactness, and phase-separated droplets each reflect distinct cellular identities, subcellular compartments, trafficking pathways, and biophysical material states. Here, I synthesize morphological signatures across neurodegenerative diseases to propose a framework that links morphology to mechanism. Morphology is neither incidental nor merely descriptive. Rather, it is a readout of the basic mechanisms that govern self-assembly of proteins into aggregates, the cell’s attempts at proteostasis (clearance, sequestration, and transport), and the failure that ensues.

## CONTACT

CONTACT (Community-Oriented Neuropathology Thoughts And Clarification Threads) facilitates interaction between neuropathology and the public. This is the response paper to: https://doi.org/10.17879/freeneuropathology-2026-9059

## Introduction

I read with great respect your meditation, “***The shapes of brain waste: Mysteries of cellular remnant morphology in neurodegeneration** (https://doi.org/10.17879/freeneuropathology-2026-9059*)”. Thank you for taking the initiative to reach out to the neuropathology community and for sharing with us your questions and musings about the various microscopic curiosities we see when studying human neurodegenerative disease tissues. Your manuscript is presented with a distinct humility as a psychiatrist without neuropathology training, and a whimsical creativity where microscopic blobs are as impenetrable as Rorschach inkblots. Morphology encompasses form, structure, and arrangement, and is a concept that cuts to the core of pathology.

**Figure 1: Convergence and Conformity.**
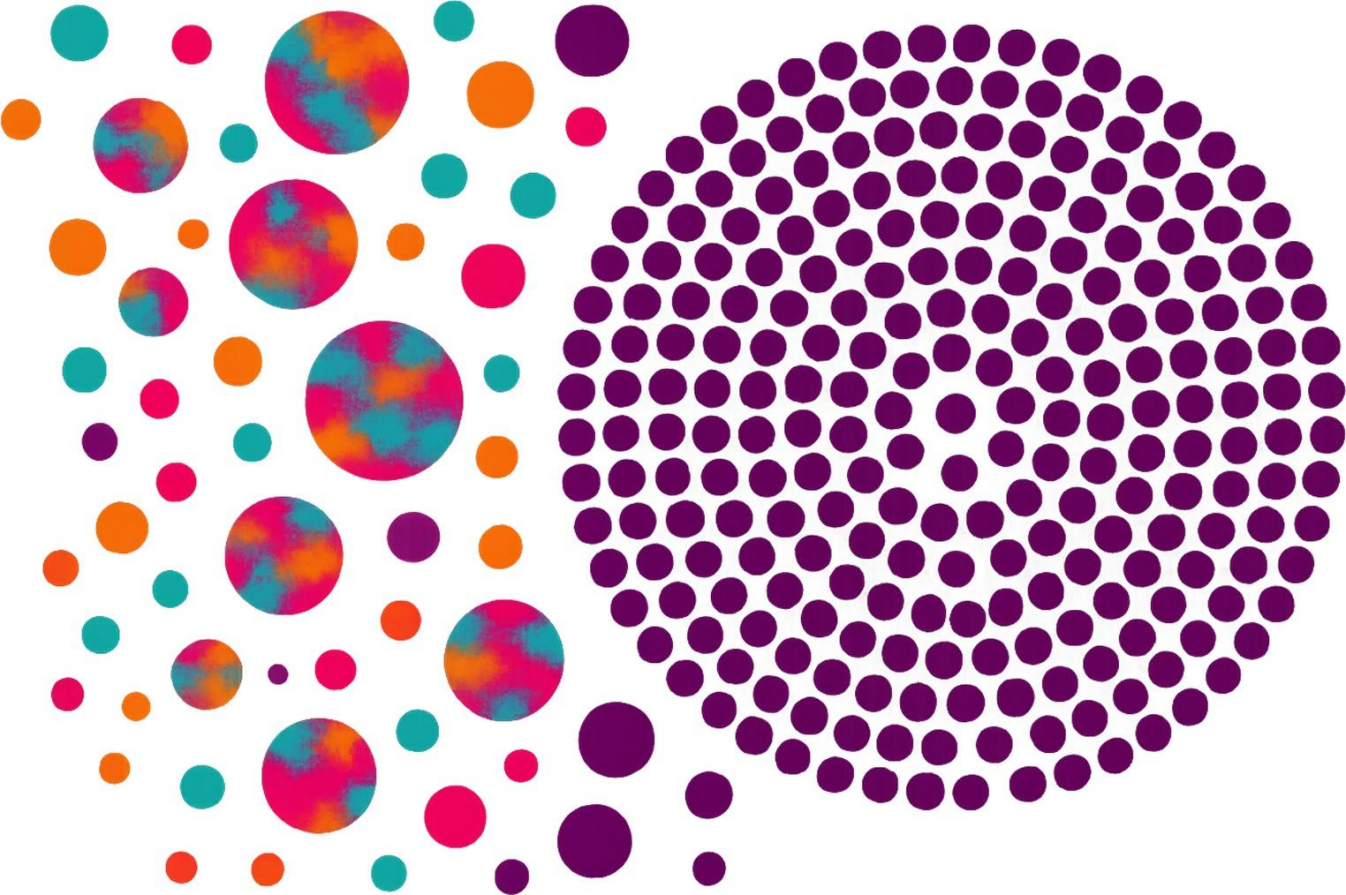
On the left, vibrant circles of varied colors and sizes represent diversity, creativity, and freedom of expression where each element stands distinct while helping form a collectively dynamic, harmonious field. Moving to the right, unique forms aggregate into an ordered cluster of deep royal purple circles: uniform, symmetrical, and rigid. There is a universal tension between complexity and uniformity whether in living systems or human societies. In the healthy brain, diverse neuronal networks interact dynamically, enabling adaptability and resilience. Neurodegenerative diseases disrupt this balance as protein aggregates accumulate, erasing complexity and imposing pathological uniformity. In social and political spheres, vibrant pluralism can give way to rigid conformity when pressures for cohesion overshadow individuality. Aggregation, be it biological or cultural, can paradoxically lead to exclusion, reminding us that true strength lies in preserving diversity within unity. Created in collaboration with artificial intelligence.

Humanity seems to have become more insular, less tolerant of those who are other, prone to co-assemble with those who are like. This self-assembly tribalism is the same process that drives the formation of protein inclusions (**Figure 1**). Ironically, pathologic inclusions are, in part, defined by the fact that they exclude other components in order to form a homotypic mass. Lest neuropathology become a homogeneous tribe, I was happy to be a reviewer of your manuscript and to suggest that we use this as an opportunity to open a dialogue between neuropathologists and those outside of our little world.

## Why morphology matters

Morphology – shape, size, boundaries, and location – provides a direct window into the cellular context of neurodegenerative pathology. It encodes which cell type is involved (e.g., neuron vs. oligodendrocyte vs. astrocyte vs. microglia), which compartment is engaged (cytoplasm vs. neurite vs. membrane-bound organelle vs. extracellular space), what transport systems are active (dynein/microtubule, actin/myosin), and what material state predominates (liquid phase-separated condensates vs. intermediate states vs. amyloid fibrils). Reading morphology as a map of mechanism can therefore guide hypothesis generation, experimental design, and therapeutic targeting.

## Rethinking the interpretation of protein aggregates

Equating protein aggregates with “dead cells” is not quite correct. The majority of protein inclusions are intracellular and occur within living cells, not mere “remnants”. Exceptions exist, such as ghost tangles and extracellular amyloid plaques, the latter representing secreted products of living cells rather than remnants of cellular death. Importantly, there is evidence that neurons harboring inclusions are not invariably destined to die. Under favorable conditions, these neurons can recover by clearing intracellular aggregates. In mice and men, it appears that treatment with antisense oligonucleotides (ASOs) which decrease expression of the *MAPT* gene by reducing new tau protein synthesis does not merely stop new tau aggregates from forming (https://www.alzforum.org/news/conference-coverage/first-hit-aggregated-tau-antisense-oligonucleotide-lowers-tangles; https://doi.org/10.1126/scitranslmed.aag0481). Rather, our neurons appear capable of clearing existing tau aggregates over time. Therefore, rather than conceptualizing cells harboring neurodegenerative disease protein inclusions as “corpses”, a more accurate characterization would be that they are “wounded”, reflecting their potential for survival and repair.

## Spongiosis and vacuoles: compartmental clues across diseases

In AD, superficial spongiosis refers to the empty spaces between neuronal and glial processes, an extracellular phenomenon tied to synaptic degeneration and swelling of organelles (mitochondria, autophagosomes) within nearby elements. The resulting neuropil resembles a cratered battlefield where remnants of cellular death and destruction produce tissue rarefaction without discrete, membrane-bounded vacuoles. This morphology implies a predominance of extracellular space secondary to disconnection of processes and neuron loss, rather than a single intracellular degradative compartment failing.

By contrast, prion diseases feature membrane-bound vacuoles within neuronal dendrites. These vacuoles often contain a few curled membranes and are enigmatic in origin (https://doi.org/10.1016/0021-9975(92)90022-m), but perivacuolar prion protein (PrP) deposits suggest a linkage to aggregate-induced responses and possibly abortive attempts at PrP clearance. The membrane-bounded nature, dendritic localization, and association with PrP argue for a trafficking and endolysosomal axis of failure, a process that culminates in focal vacuolation rather than diffuse spongiosis.

Vacuoles observed in vacuolar tauopathy (an ultra-rare frontotemporal dementia linked to a pathogenic variant in the *VCP* gene) are intracellular and morphologically consistent with abnormally swollen endolysosome-like organelles (https://doi.org/10.1126/science.aay8826). Given VCP’s established role in endosome-lysosome regulation, the vacuolar phenotype coheres with a biochemical defect in organelle quality control and trafficking.

Similarly, vacuolar annexinopathy (another ultra-rare neurodegenerative disease associated with an *ANXA11* variant) exhibits intracellular vacuoles (https://doi.org/10.1007/s00401-024-02753-7). ANXA11 binds membranes including lysosomes and is thought to tether cargoes, including RNA granules, for long-range transport in neuronal processes (https://doi.org/10.1016/j.cell.2019.08.050). The vacuolar morphology here plausibly reflects disrupted lysosome-based trafficking and cargo coupling, with vacuole formation as a structural manifestation of stalled or misregulated organelle dynamics.

Across these entities, vacuolation reflects different mechanisms of degeneration. Membrane-bound, intracellular compartments, possibly abnormal endolysosomes, are at the heart of failure modes governed by PrP-, VCP-, and ANXA11-dependent neurodegeneration, pointing towards organelle-centric dysfunction, distinct from the extracellular rarefaction of AD spongiosis.

## Oligodendroglial coiled bodies: architecture reveals identity

Certain tauopathies including progressive supranuclear palsy and corticobasal degeneration exhibit a preponderance of glial inclusions (https://doi.org/10.1146/annurev-pathmechdis-051222-120750). Notable amongst the different morphological subtypes is the coiled body, a small but readily recognizable protein inclusion comprised of tau protein. The coiled body inclusion reflects the native cytoplasmic architecture of oligodendrocytes: a small perikaryon emitting a curved process that myelinates axons. When insoluble protein fills this space, the pre-existing shape of the oligodendrocyte sculpts the inclusion and helps the neuropathologist recognize its oligodendroglial origin. In tauopathies with oligodendroglial involvement, the morphology of the coiled body is therefore not arbitrary – it is a direct imprint of cell identity and process geometry on the aggregate’s final form. Here, it is not the organelle, but the cellular architecture which dictates morphology. Similarly, coiled bodies and glial cytoplasmic inclusions (GCIs) found in multiple system atrophy are brothers-in-arms. GCIs form the same comma-shaped protein aggregate morphology but are composed of α-synuclein protein and not tau. Thus, the cell’s intrinsic architecture is one factor which governs the morphology of pathologic inclusions.

## Compartmentalizing aggregation

Although the cells within which protein aggregates form help dictate protein aggregate shapes, there are more mysterious forces at play that contribute to the subcellular localization of such aggregates. Unlike the humble oligodendrocyte with its very simple comma shape, neurons and astrocytes exhibit beautifully extended cellular processes into the neuropil ether. Hence, aggregates sometimes form within or near the perikarya (neurofibrillary tangles in AD, balloon neurons in Pick’s disease, tufted astrocytes in progressive supranuclear palsy), and sometimes form in their distal processes (tau-positive dystrophic neurites in AD, astrocytic plaques in corticobasal degeneration). Neuronal hypertrophy with perikaryal accumulation of intermediate (neurofilament) proteins is a common reactive state in injury and neurodegeneration (including axotomy models). Yet the specific path by which a neuron becomes ballooned with tau filaments – a distinctive phenotype in select tauopathies – remains unresolved. Morphology here highlights two intertwined axes: (1) cytoskeletal reorganization (microtubules, neurofilaments) that governs cell structure and bulk; and (2) the dynamic biogenesis of pathologic filaments coupled to transport, turnover, and aggregation.

While the true mechanisms that dictate the subcellular localization of many neurodegenerative disease inclusions is not exactly known, we can borrow some insights from our experimental cell biology colleagues to glean some insight. In yeast, the IPOD (Insoluble PrOtein Deposit) forms near the vacuole (a large, lysosome-like, membrane-bound, acidic compartment), collecting insoluble misfolded proteins, including amyloidogenic species, via actin/myosin-dependent vesicular transport (https://doi.org/10.1038/nature07195). These proteins are sequestered for eventual degradation, and also to avoid inheritance by daughter cells. Better for the parent to keep their own “detritus” and spare their offspring from that burden. Alas, neurons in their “post-reproductive” (post-mitotic) state cannot use the same strategy! In contrast, JUNQ (JUxta-Nuclear Quality control compartment) is a membraneless, juxtanuclear locus for soluble, ubiquitinated misfolded proteins. JUNQ is more dynamic than IPOD, but both reflect the ongoing triage of misfolded proteins by the proteostasis network (https://doi.org/10.1038/nature07195).

In mammalian cells, aggresomes form via active dynein-mediated transport of smaller aggregates along microtubules to the microtubule-organizing center, producing spherical structures encased by a vimentin intermediate filament cage (https://doi.org/10.1083/jcb.143.7.1883). This morphology indicates centralized sequestration at a transport hub, with cytoskeletal scaffolding that both contains and signals the presence of problematic cargo. While aggresomes are solid, insoluble globs of protein, not all protein assemblies are so rigid. Phase-separated granules such as stress granules tend to form spheres, with surface tension at the boundary between condensate and cytosol imparting a smooth, rounded geometry, the same dynamics that dictate the shape of rain droplets (https://doi.org/10.1074/jbc.tm118.001192). Their morphology betrays a liquid-like material state, with fusion-fission dynamics and sensitivity to RNA/protein composition, post-translational modifications, and crowding.

These experimental clues offer a mechanistic Rosetta stone for human neuropathology: spherical, membrane-less granules suggest phase separation while specific localization to different subcellular sites within neurons and astrocytes perhaps reflects abortive attempts to sequester misfolded proteins for degradation. If some aggregates are driven towards the perikarya in a desperate attempt to point them towards degradation, perhaps other cellular processes dictate the formation of aggregates out in distal cell processes? In FTLD-TDP Type C, dystrophic neurites are of dendritic origin and contain heterofilaments composed of TDP-43 and annexin A11 (ANXA11). ANXA11 is thought to tether RNA granules to lysosomes for axonal transport; while its role in dendritic RNA granule trafficking is less explored, its membrane-binding and cargo-tethering functions plausibly extend to dendrites (https://doi.org/10.1016/j.cell.2019.08.050). So perhaps the normal physiology of ANXA11 whereby it links RNA granules, some of which naturally contain TDP-43 protein, to motile lysosomes sets a dendritic stage on which TDP-43/ANXA11 heterofilaments form.

Truthfully, it would have been difficult to ascertain these potential mechanisms merely by staring at a microscopic slide. Thus, sometimes we need the help of cell biologists and an appreciation of normal cellular physiology to better understand the forces that dictate the morphology and spatial distribution of pathology.

## Amyloid plaque compactness: a glial-genetic dial

While we have so far focused on intracellular mechanisms that drive the shape of neuropathologic inclusions, there are more complicated intercellular dynamics which can also dictate morphology. The amyloid plaque in Alzheimer’s disease is a truly complex and heterogeneous structure. Fibrils made of the Aβ peptide fill the extracellular space, ensnaring neurites within their reach, neurites which accumulate tau fibrils and dysfunctional lysosomes. This complex mess of extracellular amyloid and swollen dystrophic neurites is then surrounded by reactive microglia and astrocytes in an apparent attempt to wall off the lesion.

To simplify diagnosis, the current neuropathologic criteria for Alzheimer’s disease focus on “neuritic” plaques to differentiate these pathologic structures from “diffuse” amyloid plaques which are largely regarded as innocuous (https://doi.org/10.1016/j.jalz.2011.10.007). This simplification, however, belies the plethora of different plaque morphologies we actually see under the microscope. Indeed, the classic mature “cored” plaque made famous by its appearance in the CERAD criteria tend to have only a few relatively thin dystrophic neurites in its periphery while other core-less neuritic plaques appear to harbor many more damaged dystrophic neurites. What drives plaque morphology? The type of Aβ that accumulates is one explanation, such as cotton wool plaques that are associated with certain mutations in *PSEN1* (https://doi.org/10.1007/s00401-009-0521-4). Increasingly, however, we are recognizing that the cellular response to amyloid plaques is an important determinant of plaque morphology. β-amyloid plaque compactness varies widely and likely reflects multiple factors. Secreted factors such as APOE protein, spewed by the reactive microglia reacting to amyloid plaques, help shape amyloid plaques. Indeed, coarse-grained plaques are associated with the *APOE *e4 genotype (https://doi.org/10.1007/s00401-020-02198-8). Microglial activation state, modulated by different *APOE *and *TREM2 *genotypes, appears to regulate plaque coalescence and compaction, with microglia physically intercalating and remodeling amyloid deposits (https://doi.org/10.1007/s00401-020-02200-3). The morphological endpoint thus integrates the intrinsic aggregation kinetics of Aβ with modulatory extracellular secreted factors and extrinsic glial containment.

## Structural biology and the emergence of polymorphic protein assemblies

Our journey thus far has progressed from the subcellular, to cellular, to multicellular scales. Let’s reverse course and dive deep into the tissue down to individual proteins and atoms. The rapid expansion of structural biology data has transformed our understanding of protein aggregation and its pathological consequences (https://doi.org/10.1093/jnen/nlab039; https://doi.org/10.1146/annurev-pathmechdis-051222-120750). Advances in cryo-electron microscopy, solid-state NMR, and complementary computational approaches now allow visualization of protein assemblies at near-atomic resolution. This unprecedented level of detail provides critical insights into the physicochemical forces that govern protein folding, misfolding, and self-assembly. We can now explain why certain proteins adopt filamentous architectures and identify the stabilizing interactions – such as hydrogen bonding, hydrophobic packing, steric zippers, and electrostatic complementarity – that confer remarkable resilience to these structures.

Mapping protein shape at this resolution has revealed that filament formation is not a random process but rather a highly ordered phenomenon driven by specific sequence motifs and structural constraints. These findings illuminate the mechanisms by which polymorphic aggregates arise, offering a framework for understanding the diversity of pathological inclusions observed in neurodegenerative diseases. The term “polymorphic shapes of detritus” aptly captures the structural heterogeneity of these aggregates, which can differ in morphology, stability, and biological activity despite originating from the same precursor protein. Such polymorphism is now recognized as a key determinant of disease phenotype and progression, underscoring the importance of structural studies in elucidating pathogenic pathways.

Collectively, these advances mark a paradigm shift: from descriptive observations of protein aggregates to mechanistic insights into their formation and persistence. By integrating structural data with biochemical and cellular studies, researchers are beginning to unravel how these assemblies propagate, interact with cellular machinery, and ultimately drive toxicity. This knowledge not only deepens our understanding of proteinopathies but also opens new avenues for therapeutic intervention aimed at disrupting or remodeling these stable, disease-associated structures. Yet, there is still a long way to go towards understanding how and why these unique protein filament structures relate to different aggregate morphotypes. It is fascinating to ponder why the structure of tau filaments from progressive supranuclear palsy accumulates in the proximal processes of astrocytes, while the structure of tau filaments from corticobasal degeneration accumulate at the distal ends of astrocytes. These atomic structures of various protein filaments were obtained by isolating these filaments from human brain tissue. We need to understand how these filaments fill cells *in situ* and to ascertain the mechanisms that regulate subcellular localization, the anatomic spread of pathology through the brain, and the factors that help combat these spreading spicules.

## Nanorobotic strategies for aggregate clearance and disease modification

Your invocation of “nanobots” may be deemed by some to be outlandish. However, I agree that the concept of nanorobots in molecular medicine is rapidly evolving from metaphor to reality, as researchers design targeted interventions that manipulate protein homeostasis and aggregation pathways. These approaches leverage endogenous cellular machinery or engineered molecules to act as precision tools against pathogenic assemblies. For example, our work focuses on developing VCP activators that enhance the disaggregase activity of valosin-containing protein (VCP), effectively boosting VCP’s function as a nanorobot to dissolve misfolded protein aggregates (https://doi.org/10.1172/jci169039). In parallel, other groups are advancing proteolysis-targeting chimeras (PROTACs) and molecular glues that recruit the proteasome—another cellular nanorobot—to selectively degrade aggregation-prone proteins.

Beyond proteostasis modulators, RNA-based nanorobots are being engineered to bind to and dismantle TDP-43 aggregates, a hallmark of several neurodegenerative disorders (https://doi.org/10.1016/j.neuron.2019.01.048). Immunotherapeutic strategies currently in clinical use, such as anti-amyloid antibodies, can also be viewed through this lens: they act as nanorobots that recognize and neutralize amyloid plaques in Alzheimer’s disease (https://doi.org/10.1001/jama.2023.13239; https://doi.org/10.1056/nejmoa2212948). Similarly, ASOs and small interfering RNAs (siRNAs) serve as genetic nanorobots, silencing the expression of aggregation-prone proteins and thereby eliminating the source of pathogenic structures.

These innovations underscore a critical point: while quantum computing and artificial intelligence may one day accelerate drug discovery and structural modeling, the fight against neurodegenerative disease does not hinge solely on such futuristic technologies. Instead, progress is being driven by reasoned design, empirical rigor, and sustained dedication. The convergence of structural biology, chemical biology, and RNA engineering offers tangible hope that these molecular nanorobots will reshape therapeutic landscapes and deliver meaningful clinical impact.

## A morphology-to-mechanism framework

Several principles reveal that morphology can serve as a diagnostic and mechanistic code. First, cell identity sculpts inclusion geometry. Oligodendrocyte architecture imprints coiled bodies; dendritic arborization shapes FTLD-TDP Type C neurites. Second, compartmentalization and transport vectors determine boundary conditions and guide localization. Membrane-bound vacuoles (endolysosomes) contrast with extracellular spongiosis; dynein/microtubule centripetal flow yields aggresomes; actin/myosin drives perivacuolar IPOD; lysosome-coupled motility (ANXA11) sets neuritic routes. Third, material state governs shape. Amyloid fibrils generate rigid, compact aggregates; liquid-liquid phase separation yields spherical, dynamic granules; intermediate states can mature or arrest, producing signature morphologies. Fourth, glia remodel pathology. Microglial states modulate plaque compactness and neuritic containment; astrocytes and oligodendrocytes contribute distinct inclusion types and extracellular milieu. Finally, proteostasis factors shape aggregates. Neurodegenerative diseases are characterized by the inability of our endogenous proteostasis network to resolve protein aggregates. Efforts to target proteostasis pathways have the potential to shape how we treat neurodegenerative diseases.

## Conclusions

Neurodegenerative pathology is sculpted by cell type, compartment, transport, material state, and proteostasis. Morphology is thus a mechanistic readout, not a passive descriptor. Admittedly, there is a lot that is unresolved and unknown. However, I feel blessed to be a neuropathologist as I can incorporate knowledge of morphology in an attempt to infer mechanism. My job as a neuropathologist is to recognize morphology-to-mechanism relationships not only to reach a diagnosis but to inform disease pathophysiology. Admittedly, many of these are obscure codes that challenge even the most resourceful detectives to extract a modicum of disease pathophysiology, but morphology allows me to form a theoretical framework from which more experimental approaches can transform correlation into causation.

## Conflict of interest statement

The author declares no conflict of interest.

